# An entropic measure of diverse specialization highlights multifunctional neurons in annotated connectomes

**DOI:** 10.1162/NETN.a.533

**Published:** 2026-03-20

**Authors:** Sung Soo Moon, Lidia Ripoll-Sánchez, Petra Vértes, William R. Schafer, Sebastian E. Ahnert

**Affiliations:** Department of Chemical Engineering and Biotechnology, University of Cambridge, Cambridge, UK; Neurobiology Division, MRC Laboratory of Molecular Biology, Cambridge, UK; Department of Psychiatry, Cambridge University, Cambridge, UK; Department of Biology, KU Leuven, Leuven, Belgium; Alan Turing Institute, London, UK

**Keywords:** Connectome, Annotated networks, Entropy, Drosophila melanogaster, C. Elegans, Information flow

## Abstract

The creation and curation of synaptic-level neuronal networks, or connectomes, enables the study of the relationship between neuronal structure and function. Topological characteristics of neuronal networks have been studied extensively. Separately, there have been considerable efforts to classify the morphology, cell types, and lineages of neurons. Here, we introduce a network metric that combines topological analysis with node metadata. This entropic quantity measures the diversity of incoming or outgoing connections to a node in terms of the metadata distribution. We find that in *Caenorhabditis elegans*, the top-scoring neurons (PVR, RMGL/R, DVA, CEP, ADE, URXR, RIGL, BAG, SMBDL) have known functions that integrate and disseminate multimodal information involved in sensorimotor functions. In the nerve cord of *Drosophila melanogaster*, we find that top-scoring neurons are embryonic neurons located in the abdominal neuropil, where sensorimotor coordination is required for complex innate behavior such as mating.

## Author Summary

Most brain network data sets are endowed with a catalogue of annotations that supplement the anatomical connectivity. Annotations grant additional interpretability in conjunction with network analysis, and methodology incorporating metadata have been understudied. We introduce a measure based on information entropy to quantify a given neuron’s level of diverse specialisms of its partners. Here, we consider anatomical and developmental annotations for the *C. elegans* and the *Drosophila* nerve cord connectomes. Our results verify prior experimental findings in identifying neurons that integrate and disseminate specialized channels of annotation connectivity.

## INTRODUCTION

A brain is often modeled as a network (Bassett & Sporns, 2017; Bullmore & Sporns, 2009), but how are brain networks organized? Neuronal resolution synaptic mapping of the brain, or connectomes, are networks of nodes and weighted, directed edges between them. While the *C. elegans* connectome (White, Southgate, Thomson, & Brenner, 1986) was a landmark data set in neuroanatomy, we have yet to formulate a general network-level understanding of the organizational principles of nervous systems. More recently, the adult *Drosophila melanogaster* brain (Dorkenwald et al., 2024; Scheffer et al., 2020; Zheng et al., 2018) and nerve cord (Cheong et al., 2024; Court et al., 2020; Marin et al., 2024; Stürner et al., 2024; Takemura et al., 2024; Venkatasubramanian & Mann, 2019), data sets have been imaged, segmented and reconstructed, and annotated. With high-throughput experimental techniques granting more metadata about the nature of the neurons (Harris, Pfeiffer, Rubin, & Truman, 2015; Janssens et al., 2022) and the synaptic connections (Buhmann et al., 2021; Cook et al., 2019; Eckstein et al., 2024; Varshney, Chen, Paniagua, Hall, & Chklovskii, 2011), connectome data sets are complex annotated systems and not simple undirected networks. Annotations are key supplements to our understanding of brain structure and function, often related to dynamical properties of the system, and intrinsic to biological function (Bazinet, Hansen, & Misic, 2023; Bazinet, Hansen, Vos de Wael, et al., 2023; Randi, Sharma, Dvali, & Leifer, 2023). However, current methodologies often overlook them; extensions of network methodologies respecting annotation metadata in neuroscience are thus needed to bridge the gap between brain structure and function.

The application of network analysis to neuroscience has proved fruitful, establishing key interpretable features of brain networks, including small-worldness, modularity, hubs, rich-clubs and community structure (Bassett & Sporns, 2017; Betzel, Puxeddu, & Seguin, 2024; Bullmore & Sporns, 2009; Jarrell et al., 2012; Towlson, Vértes, Ahnert, Schafer, & Bullmore, 2013). Many analyses focus on assigning an importance to a node in the context of global topology, such as centrality measures. However, measures based on local topology may be more appropriate for studying biological networks with specific cross-modular functional roles. For instance, network motifs have been shown to form the basis of many complex networks (Milo et al., 2002), especially in brain networks where motif analysis revealed that complex behavior emerges from local circuit building blocks (Sporns & Kötter, 2004). Topological network metrics have been used in combination with metadata in some contexts, such as assortative mixing (Bazinet, Hansen, & Misic, 2023), but previous studies on network motifs in single-neuron resolution data in *C. elegans* (Bentley et al., 2016; Milo et al., 2002; Varshney et al., 2011) and *Drosophila* (Hulse et al., 2021; Lin et al., 2024) do not fully exploit the rich metadata available. Comprehensive brain networks are key to understand principles of development (Schlegel, Costa, & Jefferis, 2017), while interpreting local connectivity can illuminate the architectural blueprint of the brain (Vogelstein et al., 2019). In this work, we introduce a method for exploring local connectivity with annotations.

As the number of available connectomes continues to increase, we require more network methods that incorporate the rich complexity of annotations to link the topology of the network to its biological function. Here, we focus on node metadata to introduce a measure of the diversity of local connections and apply this to the *C. elegans* connectome and the *Drosophila* ventral nerve cord (VNC) connectome. The *C. elegans* dataset provides the connectome of a complete nervous system, whereas the *Drosophila* nerve cord offers an interpretable model in which information flows between well-defined inputs and outputs with less complexity than the brain (Cheong et al., 2024). We exploit several different annotation schemes, from fine-grained labels such as cell types to broader categories such as lineages. While there is prior work characterizing diversity with respect to community annotations (Bertolero, Yeo, & D’Esposito, 2017; Delaplace, 2025; Eagle, Macy, & Claxton, 2010; Guimerà & Amaral, 2005; Guimerà & Nunes Amaral, 2005; Pedersen, Omidvarnia, Shine, Jackson, & Zalesky, 2020) and information entropy in network science is not novel (Bashiri, Rahmani, Bashiri, Módos, & Bender, 2020; Omar & Plapper, 2020), to our knowledge, no prior work aims to characterize the diversity of specialized connectivity in the local neighborhood of a given node.

**Figure 1.**
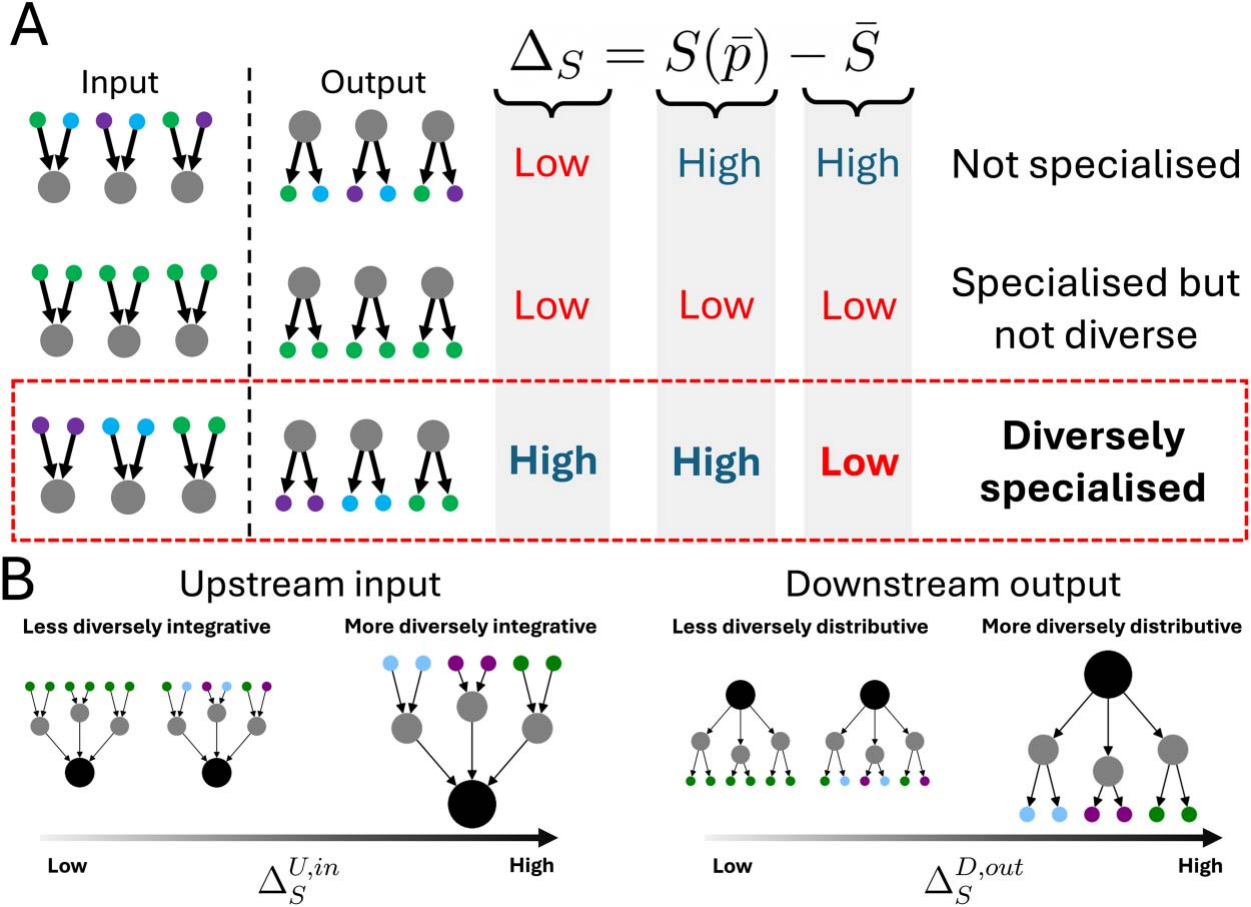
A measure for quantifying diversely specialized connectivity vectors. (A) Here, we illustrate three scenarios concerning input or output connectivity vectors of the three gray nodes (left), where the colored partners represent different annotation types. The value of entropy is low when the probability distribution is most specialized in one category and highest when it is evenly distributed. In the first row, the individual vectors are not specialized, leading to a high average entropy, $\bar{S}$ and a high entropy of average probability vector $S\left(\bar{p}\right)$. In the middle row, the three gray nodes are specialized in the same annotation type. Therefore, the average entropy is low and the entropy of the average vector is also low (in this case, $\bar{S}=S\left(\bar{p}\right)=0$). For the above reasons, the two cases yield a low value for Δ_*S*_. In the last row, the gray nodes are also individually specialized in their vectors. In contrast to the previous case, the gray nodes are specialized in different types. Therefore, while the average input entropy is low again, the average input $\bar{p}$ is not specialized and so $S\left(\bar{p}\right)$ is high. Thus, the difference, Δ_*S*_ is only high when the gray nodes are specialized diversely. We invite readers to the Supporting Information for a mathematical comment on the bounds of possible Δ_*S*_ values. (B) We extend the previous argument to two motifs to calculate the specialization-diversity, and illustrate the cases where the Δ_*S*_ values are low and high. Firstly, with the black node’s upstream partners’ input vectors we measure the integrative specialization-diversity $\Delta_{S}^{U,\text{in}}$ (left). Secondly, the specialization-diversity of the downstream partners’ outputs measures the distributive specialization-diversity $\Delta_{S}^{D,\text{out}}$ (right).

Our measure, the *specialization-diversity*, is derived as follows: first, we construct each neuron’s connectivity vector *p*, which expresses the proportion of incoming or outgoing connections of that neuron to other neurons. The metadata we use are cell classifications or lineage annotations (see Methods section for details). Then, for a set of connectivity vectors {*p*}, the specialization-diversity is defined as: $\Delta_{S}=S\left(\bar{p}\right)-\bar{S}$ where *S* = −∑*_i _p_i_ ln* (*p*_*i*_), information entropy and $\bar{p}$ and $\bar{S}$ denote the means of *p* and *S*. A large Δ*_S_* value is only possible when the vectors {*p*} are individually specialized but in diverse categories (see Figure 1A and Methods). Δ*_S_* quantifies the level of diverse specialization in connectivity with respect to the annotations present, and this is a property of the set of connectivity vectors. We consider two sets of vectors for a given neuron that characterize the diversity of its integrative and distributive connectivity. The first is calculated from the diversity of specialization in the downstream outputs, and the other is calculated from the diversity of specialisation in the upstream inputs. More precisely, for a target neuron, we gather *out*going connectivity vectors of *downstream* partner neurons, and calculate a specialization-diversity. The resulting Δ*_S_* associated to this outwards branching motif is what we call the *distributive specialisation-diversity* and notate it as $\Delta_{S}^{D,\text{out}}$. Similarly, a given node’s *upstream* partners’ *in*coming vectors can be used to calculate a specialization-diversity. We call this *integrative specialization-diversity*, $\Delta_{S}^{U,\text{in}}$. These measures quantify the level of diversely specialized connectivity channels in a node’s local vicinity (Figure 1B), with respect to an annotation scheme we use to label the nodes. The annotations we used for the connectivity vectors were cell categories in *C. elegans* and hemilineage in *Drosophila*. These annotations are anatomical and developmental descriptors that are correlated to function and behavior, and with our measure, we are quantifying the most diversely specialized neurons by these associations.

In this paper, we interpret the diversity of local connectivity as a characteristic of the role of a node in the connectome by using the specialization-diversity measure. The top 10 neurons with the highest summed integrative and distributive specialization-diversities we call “entropic hubs.” In *C. elegans*, we find these to be linked to multimodal functions having roles in sensorimotor circuits, and in neuropeptide signaling networks (Ripoll-Sánchez et al., 2023). In the *Drosophila* nerve cord, our entropic hubs are concentrated in the abdominal region where higher-level innate behavioral programs are controlled (Cheong et al., 2024; Pavlou et al., 2016).

## RESULTS

### Cell Classes Occupy Interpretable and Distinct Regions in Specialization-Diversity Distributions

We constructed vectors that aggregated the connectivity to the annotations for each neuron. In *Drosophila*, we used hemilineage annotations, which are developmental metadata and have been experimentally associated with locomotive functions like walking and flight (Harris et al., 2015). For *C. elegans*, there have been extensive efforts in cataloguing the neuronal types we may use as metadata. The finest level of classifications were the from gene expression profiles splitting 302 neurons into 118 types (Taylor et al., 2021). Coarser cell classifications came from identifying broad neuronal function: sensory neuron, motor neuron, pharynx, and interneuron classifications. There are “mid-scale” classifications that are based on anatomical position, specific sensory modality or motor program as well as information flow (Cook et al., 2019). We chose the mid-scale cell category to construct the vectors that were associated with specialized functions, for instance, SN3 refers to mechanosensory neurons. Additionally, the mid-scale annotations offered a balance such that the connectivity vector would not be too distinct for similar neurons, and conversely, coarse-grained annotations are too general (see Methods section and Supporting Information for a breakdown of neurons into their cell classifications). We reserved the finest and broadest cell type classifications for validation with null models.

**Figure 2.**
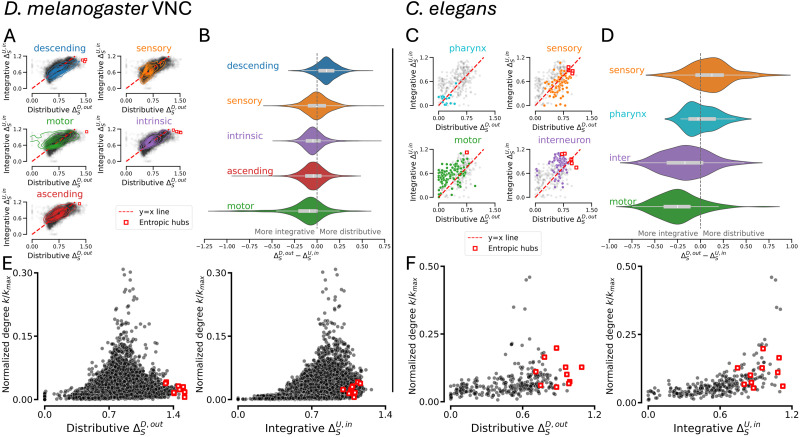
Specialization-diversity distributions are localized for different cell classes, and are distinct from synaptic degree. The distributions of integrative versus distributive specialization-diversity for *Drosophila* VNC neurons (A) and *C. elegans* (C) show localization of the cell class populations in the plot, with transparent black markers showing the full distribution. In dotted red is the y = x line where the neuron is equally distributive and integrative. Red squares are entropic hubs: neurons that rank in the top 10 highest values of $\Delta_{S}^{D,\text{out}}+\Delta_{S}^{U,\text{in}}$. The distribution of distributive minus integrative specialization-diversity for all neurons in the *Drosophila* VNC (B) and *C. elegans* (E), reveals that all cell classes have members that are strongly distributive and or integrative, but the majority are well balanced. The averages of the distributions are sorted from most distributive (compared with integrative) at the top to most integrative (compared with distributive) at the bottom. The cell classes are appropriately biased corresponding to their functional role. If the role is primarily projective into the system, then it is more distributive than integrative (descending, sensory). In *Drosophila*, the sensory neurons are more balanced as the distribution mean lies around zero, suggesting that the average neuron will be equally integrative and distributive. If the role is primarily integrating diverse channels of information, then outputs in specific and specialized ways, the class is more integrative than distributive (motor, ascending). Intrinsic neurons in *Drosophila* and interneurons in *C. elegans* have a skewed mean toward integrativeness suggesting the function requires more integration of diverse connectivity than distribution. Subfigures E and F show that our measure is distinct of degree, the number of total partners of a node. In both animals, the entropic hubness cannot be fully explained by sheer number of synaptic partners, as neurons with the highest specialization-diversity values occupy low (normalized) degree values.

We first verified whether the specialization-diversity was interpretable when considering broad cell classifications in the two connectomes by inspecting the distributions for integrative and distributive diversities (Figures 2A for *Drosophila* VNC and 2D for *C. elegans*). In both distributions, neurons are broadly speaking similarly distributive and integrative, but nevertheless there are clear deviations that differ according to the cell classes. In Figures 2B and 2E, we show the distributions of the quantity $\Delta_{S}^{D,\text{out}}-\Delta_{S}^{U,\text{in}}$, which quantifies how much more distributive or integrative a given neuron is. We report varying biases between the cell types—sensory (orange) being most distributive, and the intrinsic/interneurons (purple) and motor neurons (green) being more integrative. In both *Drosophila* and *C. elegans*, sensory neurons with mostly downstream connections will propagate into the connectome for the processing of external sensory signals, and so are biased toward being distributive rather than integrative. Motor neurons, by contrast, will mostly form upstream connections that control their output, and therefore are more integrative than distributive. The ascending (red) and descending neurons (blue) in *Drosophila* lie in the distributions as we expect: descending neurons being more distributive and ascending more integrative. For intrinsic neurons in *Drosophila* and interneurons in *C. elegans* that mediate the connectivity between the inputs and outputs of the system, we see a slight bias toward integrative diversities. The pharynx (teal) in *C. elegans* is very sparsely connected to the rest of the network and therefore exhibits low distributive and integrative diversities.

### Entropic Hubs Are Not Synaptic Hubs

As the distributions of the broad classifications of neurons yield results we can intuitively interpret, we now consider finer classifications and individual neurons. Firstly, we consider the highest-ranking neurons in $\Delta_{S}^{U,\text{in}}+\Delta_{S}^{D,\text{out}}$ value, which we call “entropic hubs” (red squares in Figure 2). In *Drosophila*, the cell classes of the 10 highest-ranking entropic hubs are three descending neurons, one ascending neuron, five intrinsic neurons, and one motor neuron, and all these neurons are more distributive than integrative. These neurons have not been previously described in detail individually, but we note that all of them have soma positions in the abdominal neuropils, which are shown to contribute to complex mating behavior (Marin et al., 2024; Pavlou et al., 2016). The entropic hubs here are likely all primary (embryonic) neurons (seven labeled and three to be further studied). Primary neurons themselves do not yet have an associated lineage in the metadata, but see Supporting Information for analysis of the neurons that have hemilineage information and associated behavioral phenotypes. In *C. elegans*, the entropic hubs play an active role in the neuropeptidergic network (see below). In both connectomes, we find that entropic hubs are not synaptic hubs (Figures 2C and 2F), meaning that a high number of synaptic partners does not result in a highly diverse set of upstream or downstream neighbor specializations. This highlights that our method focuses on the specificity of connectivity rather than the volume of it. The full distributive and integrative specialization-diversity distributions of all neurons and their metadata are made available (see Methods section).

### Neurons Within Fine-Grained Annotation Types Are Similar in Terms of Their Specialization-Diversity Values

The finest level of annotation in the connectome data sets are cell types. In *C. elegans*, this comprises 118 cell types that are characterized and named according to their morphology and position. In the *Drosophila* VNC, we have cell types that group neurons by identical cell morphology or connectivity. Nervous systems exhibit a well described bilateral left–right symmetry observed in the connectome (Cheong et al., 2024; Marin et al., 2024; Venkatasubramanian & Mann, 2019; Winding et al., 2023). In the *Drosophila* VNC, due to the sixfold symmetry of the leg neuropils, we have six identical analogous neurons forming one *serial homologue* group (Haszprunar, 1992; Marin et al., 2024). These neurons are assigned the same label, but are not necessarily strongly connected, so do not form a modular structure within the connectome. Rather, these neurons are similar in morphology, so they have similar connectivity within each modular structure.

**Figure 3.**
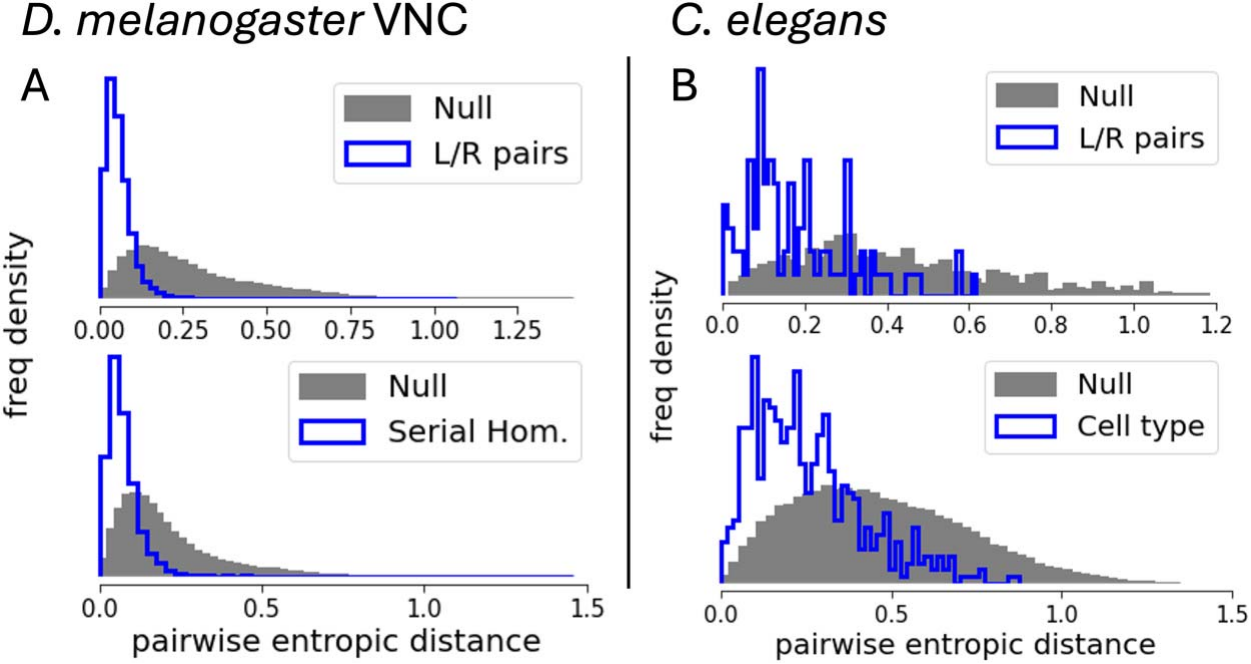
Cell types, left–right pairs, and serial homologues are statistically significant in their specialization-diversity values. The figure shows statistical significance of the specialization-diversity values for different types of fine-grained neuron categorization. In all cases, we calculate pairwise Euclidean distances of the specialization-diversity vector $\left[\Delta_{S}^{D,\text{out}},\Delta_{S}^{U,\text{in}}\right]$ within a specific category and compare this with an appropriate randomized null model. We call this distance the pairwise entropic vector distance. In the VNC (A), left–right pairs (top) and serial homologues (bottom) are demonstrated to be statistically significant in their specialization-diversity values. For left–right pairs, we construct a null model by randomly generating pairs of neurons that conserve the side of the soma, that is, each randomized pair would be from opposite halves of the VNC. The serial homologue null model picks random groups of six neurons but conserves the leg neuropil membership, that is, six random neurons from each leg neuropil. In all null models, we preserve the connectivity vector and thus the entropy vector. We find qualitatively identical results for *C. elegans* (B) using analogous distance calculations using appropriate null models for statistical significance of left–right pairs (top) and fine-grained cell types (118 types of which left–right pairs are a subset). The left–right null model again conserves the side membership, and the null model for cell type uses an all-by-all distance calculation (see main text). All null model results are aggregated over 10 realizations of random sampling. Two-sample Kolmogorov–Smirnov tests yielded significant results for all distribution comparisons with their nulls (*p* << 10^−3^).

We asked if these finer groups of similar neurons were similar in terms of their specialization-diversity, which was calculated from connectivity aggregation of the mid-scale cell categories for *C. elegans* and hemilineage for *Drosophila*. To answer this, we calculated pairwise distances within the fine groupings using the Euclidean distance on the embedding space of the distributive and integrative diversities $\left[\Delta_{S}^{D,\text{out}},\Delta_{S}^{U,\text{in}}\right]$. Explicitly, the Euclidean distance between neurons *i* and *j* is $d_{ij}=\sqrt{{\left({\left(\Delta_{S}^{D,\text{out}}\right)}_{i}-{\left(\Delta_{S}^{D,\text{out}}\right)}_{j}\right)}^{2}+{\left({\left(\Delta_{S}^{U,\text{in}}\right)}_{i}-{\left(\Delta_{S}^{U,\text{in}}\right)}_{j}\right)}^{2}}$. We then tested whether this distribution deviated from appropriate null models (Figure 3). We report statistically significant similarity of contralateral (left–right) pairs using a side-preserving null model of random samples of two neurons, one from each lateral side (top row, Figure 3). We also found statistically significant similarity within serial homologue groups in *Drosophila* (Figure 3A, bottom), where the null models are pairwise distributions drawn from sampling one neuron from each leg neuropil. Lastly, we tested for pairwise entropic distance within within the 118 cell types in *C. elegans* (Hobert, Glenwinkel, & White, 2016) (Figure 3B bottom), with a null model that considers the all-neuron-by-all-neuron pairwise entropic distance distribution. Our results show that similar neurons have similar values of integrative and distributive specialization-diversities to a statistically significant degree according to a two-sample Kolmogorov–Smirnov test. By testing our null models with the finest-grained cell classifications we have validated our method with the strictest possible criteria.

### *C. elegans* Entropic Hubs Have Active Roles in Complex Behavior

Since all neurons in *C. elegans* are named and have been subject to (in some cases extensive) experimentation, we describe the entropic hubs and their active roles in complex behavior. The top ranking neuron, PVR, is a tail interneuron not well described in function. Recently, PVR has been identified as a neuropeptidergic network hub, important in the “wireless” neuropeptide-GPCR (G protein-coupled receptor signaling complementary to the synaptic and gap junction “wired” network (Ripoll-Sánchez et al., 2023). PVR was also predicted to be a motor control neuron (Yan et al., 2017). Neuron class RMG (RMGL and RMGR) are interneurons described in the literature as an essential neuropeptide expressor responsible for all types of social behavior, whose complex behavior depends on many modes of sensory cues and many diverse repertoire of outputs, with RMG being a gap junction hub for connecting seven classes of neurons (Macosko et al., 2009). Neuron DVA is a stretch sensory neuron for proprioception, and positively and negatively affects locomotion to be able to fine-tune motor neuron stimulation, from a single source. Its central role in regulating the sensory-motor integration explains the diverse downstream specialism (Li, Feng, Sternberg, & Shawn Xu, 2006). DVA is also an important neuropeptidergic hub (Ripoll-Sánchez et al., 2023) and a hub node linking multiple layers of signaling (synaptic, neuropeptidergic, and monoamine) in *C. elegans* (Bentley et al., 2016). The CEP neurons are dopaminergic touch sensory neurons in the nose (Kang, Gao, Schafer, Xie, & Xu, 2010; Kindt et al., 2007) responsible for food slowing (Sawin, Ranganathan, & Horvitz, 2000) and, along with ADE, other behavioral responses to food (Chou, Chen, Liao, & Pan, 2022; Ezcurra, Tanizawa, Swoboda, & Schafer, 2011; Ezcurra, Walker, Beets, Swoboda, & Schafer, 2016). Neuron URXR is a body cavity oxygen sensory neuron, central in homeostatic integration of oxygen level fluctuation and metabolic processes. Its sensory signals stimulate body fat loss only when the body has sufficient reserves (Witham et al., 2016). Neuron RIGL is a ring interneuron part of four pairs of neurons that mediates CO_2_ response, operating downstream of BAG oxygen sensory neurons. Hunger causes the animal to be attracted to CO_2_, and feeding reverses this, and neuron pairs RIG and AIY mediate this (Rengarajan, Yankura, Guillermin, Fung, & Hallem, 2019). Neuron SMBDL is a dorsal neck motor neuron that is a part of the SMB cell type that is crucial in sensorimotor transformation for governing klinotaxis, or stimulus provoked lateral locomotion, in particular, changes in salt concentration (Matsumoto et al., 2024).

## DISCUSSION

Our objective in this study was to incorporate biological annotations to enhance our understanding of functional roles in structural brain networks. To achieve this, we have introduced a novel measure based on information entropy that describes the level of diverse specialization in local directed node neighborhoods that are integrative and distributive. The specialization-diversity considers aggregated, directed connectivity from next-nearest network neighbors, thus, it is a localized measure for a given node that does not rely on global knowledge.

The application of our approach to the *C. elegans* and *Drosophila* VNC connectomes, using cell classification annotations and hemilineage annotations respectively, yielded distributions of specialization-diversity that reflect the biological functionalities of these neuron classes. If a given target neuron is on any directed path of length two as the source or sink of that path, then these quantities can be calculated. Therefore, almost all neurons can be quantified by their proximity to specialized connectivity patterns. The capability of neurons to integrate and broadcast the many modes of diversely specialized information aligns with the roles of cell classes. However, all cell classes have outliers that are on either extremes, suggesting heterogeneity and hierarchy in diverse specialization within class modalities in nervous systems. Interestingly, intrinsic neurons and interneurons that form the bulk of the connectivity bridging inputs and outputs of the network are more integrative than distributive on average. This asymmetry suggests that their connectivity role is to integrate diversely specialized channels of annotation types. Specific entropic hubs with highly integrative and distributive connectivity channels are neurons that are responsible complex sensorimotor behavior in *C. elegans*, thus requiring close proximity to specialized channels in their inputs and outputs. We propose that entropic hubs in the *Drosophila* VNC are responsible for sensorimotor behavior, and previous experimental work on the abdominal neuropil suggests an important role in innate behaviors like mating (Pavlou et al., 2016), and so we expect this result to be qualitatively conserved across different individuals.

While we can identify top entropic hubs that are most integrative and distributive, we are limited in terms of the conclusions we can draw from this in *Drosophila*. We have chosen the VNC as a model data set for our analysis, because of its clarity of input and output modalities (Cheong et al., 2024). With a relative simplicity of sensory and executive brain inputs to motor and feedback into brain outputs, the structure presents an ideal test case that might be difficult to disentangle for more complex data sets, such as the brain. We also justify that for the VNC, the hemilineages are strongly linked to motor functions and processing, and analogous conclusions would not be made if similar annotations were not available in other data. The VNC facilitates both reflexive and coordinated movement in the fly independent of brain signaling, the network topology is rich with functional circuitry that allows for complex behavior (Takemura et al., 2024). Secondary (postembryonic) neurons of specific hemilineages have been shown to relate to specific motor functions (Harris et al., 2015), but in our analysis, we found the specialization-diversities to vary between hemilineages annotated with the same function, suggesting further sub-roles, requiring different levels of diversely specialized connectivity (see Supporting Information). Our Δ_*S*_ indirectly measures the neurons that are proximal to many diversely specialized hemilineage connectivity channels responsible for coordinated movement like walking and flight. The entropic hubs in *Drosophila* are mostly primary (embryonic) neurons (seven characterized as such and three not yet annotated). Primary neurons are often globally and centrally connected, and more likely be a member of rich club organization (Marin et al., 2024), possibly facilitating the high integrative and distributive specialization-diversities. Early birthtime neurons go through a developmental program that persists and are “remodelled” during metamorphosis into the adult (Marin et al., 2024; Shepherd, Harris, Williams, & Truman, 2016), and we speculate that its proximity to highly diversely specialized connectivity are artefacts of dynamic utility and versatility pivotal during development. The entropic hubs are associated with complex, often sensorimotor behavior in *C. elegans*, with some neurons that were only recently described to be significant like PVR, a neuropeptide hub (Ripoll-Sánchez et al., 2023). We also report that entropic hubs are not conventional hubs in terms of their degree in the synaptic network. In *C. elegans*, while some entropic hubs have high degrees compared with the rest of the network, there are several neurons that exhibit highly distributive and integrative diversities without being synaptic hubs. In *Drosophila*, the absence of correlation between specialization-diversity and degree is clearer. We observe that while highly connected nodes could also be in close proximity to highly specialized and diverse connectivity channels, their high degree makes them more likely to be connected to the same specializations, and nonspecialized partners, which penalizes their specialization-diversity value. This is consistent with previous studies that showed that node degree was independent of a node’s diversity of connection to disparate modules (Pedersen et al., 2020).

We have verified that the cell types, contralateral pairs, and serial homologues are more similar to each other in specialization-diversity values than in randomized null models. Thus, similar neurons are also similarly diversely connected. This is expected as cell types are often defined to be an irreducible subunit of neuronal grouping (Dance, 2024; Hobert et al., 2016; Schlegel et al., 2024), thus are believed to be functionally identical and so identically connected. The statistical significance of the pairwise entropic distances against various null models of cell types and serial homologues provides comparative methodologies crucial for cross-identifying and contrasting different connectome datasets (Schlegel et al., 2024; Stürner et al., 2024). However, the specialization-diversity values of functionally different neurons overlap considerably. While we are able to recover the broad cell classifications to be localized at low or high integrative or distributive specialization-diversity values, it is insufficient to infer the specific finer-grained categorizations from specialization-diversity alone. Since we have reduced the network of annotated nodes into the two numbers quantifying the proximity to diversely specialized channels of connectivity, equivalently diverse connectivity may arise from different connectivity specialization. We leave this inverse inference problem for future work, because approaches like graph neural networks (Corso, Stark, Jegelka, Jaakkola, & Barzilay, 2024) have demonstrated convincing evidence that node annotations supplement our inference of function in the underlying network. We also speculate more open questions about the nature of biological annotations complementing the network connectivity. Many different annotation schemes are available from coarse to fine-grained; node or edge metadata and their informative or predictive relationships with network structure are not fully understood (Bazinet, Hansen, & Misic, 2023; Bazinet, Hansen, Vos de Wael, et al., 2023). While our methodology can be extended to handle these, we do not yet know of which annotation schemes are most biologically and inferentially complementary for detecting specific functional attributes of the underlying system.

The connectomes we have used here also have limitations, for instance, in terms of the incompleteness of annotations in *Drosophila*. Hemilineage annotations are inferred from morphology and connectivity and are incomplete for primary (embryonic) neurons, which are functionally and morphologically distinct from secondary (postembryonic) neurons. We ignore connectivity with unlabeled nodes in our methodology, but expect our conclusions to remain qualitatively robust since primary neurons arguably should be treated as a separate hemilineage subtype in our methodology because their functions and morphology are distinct to secondary neurons of the same lineage. Hemilineages are developmentally driven functional units of the nerve cord; thus, we expect the average connectivity statistics to be conserved across individuals, as demonstrated in recent comparative studies (Lin et al., 2024; Schlegel et al., 2024; Stürner et al., 2024). Moreover, the *C. elegans* connectome has been shown to have high conservation of connectivity across individuals and developmental stages (Cook et al., 2019; Witvliet et al., 2021); thus, we expect our results to conform on average with new data. Another limitation of the work is that our results are speculative and predictive rather than conclusive of function or activity, which has been described in anatomical perspectives of neuroscience previously (Prinz, Bucher, & Marder, 2004). Since our specialization-diversity pools connectivity from all neurons that are reachable by or with a directed path of length two, we are compounding biological variability, or errors attributed to connectome imaging and reconstruction present in the data. Despite this, our analysis shows sufficient evidence that aggregating by connectivity produces statistically significant results to justify this study, and so expect our conclusions be qualitatively similar with future data that arises from advances in the acquisition of connectomic data. We expect future connectome data sets to be accompanied by a variety of annotations, from morphology to genetic annotations. Annotations are likely to be a key aim of synapse resolution connectomics pipelines to catalogue the neural inventory that informs other neuroscientific fields. This paper serves as a proof of principle of our method that is shown to provide interpretable insights in connectomes across two different magnitudes. This indicates that our specialization-diversity can be straightforwardly applied to larger and more complex data sets, like the full *Drosophila* brain (Dorkenwald et al., 2024).

Our measure aggregates the connection profiles of neurons that are reachable by a path of length two from a given query neuron, and compares their specialization. There are opportunities to extend this measure for interesting metrics quantifying the diversity of connectivity. The base methodology can take any set of normalized probability distributions to quantify the diversity of the specialization. An opportunity for future work exists to extend the method to consider specific circuits in the network and calculate the specialization-diversity of neurons along a path. These path-based measures could quantify the distinct annotation profiles that could exist in a query signaling pathway, where the extension of the metric may allow comparisons of the extent of diverse integration or distribution of these query pathways. Additionally, inspecting the specific annotations that contribute to the pathway scoring highly in a specialization-diversity measure may elucidate the specific modalities contributing to the information processing along the path. For instance, a touch-sensory to a body motor neuron pathway may exhibit a repelling or attracting behavior depending on other sensory cues.

Data on the large-scale organization of nervous systems has only recently become available to the neuroscientific community. Our primary aim was to provide an augmented perspective of network analysis that incorporates node annotations. To do this, we introduced an entropy-based measure, demonstrated its potential as an interpretable descriptor of neuronal function, and proposed roles for neurons not yet individually characterized experimentally. More generally, the specialization-diversity is a local centrality measure in annotated networks that can propose key nodes that are important for aggregating and disseminating diversely specialized channels of connectivity. Similarly, this structural identification of the possible integration and distribution of annotation connectivity can be expanded to other connectome types. With a careful choice of annotations, the method could reveal diversely integrative and distributive regions. Nodes with diverse connections but not necessarily large numbers of connections are especially interesting in neuroscience (Bertolero et al., 2017; Pedersen et al., 2020), and our methodology requires high coverage of meaningful annotations to uncover these structures. Other examples of useful annotations are transcriptomic profiles that catalogue the molecular functionality in each region, ideal for meso-scale connectomes without general single-neuron knowledge to characterize functionality. However, we do not expect to exactly retrieve our results for networks of different resolution, as by construction, the complexity of connectivity that our method relies on is averaged out. While we have demonstrated our method using neuronal connectomes, it can therefore be applied to any complex network with node annotations.

## METHODS

### Specialization-Diversity

To calculate the specialization-diversity, we require normalized connectivity vectors describing the proportion of connectivity of a given neuron to each annotation label. We first establish the labels for all nodes we adhere to for connectivity aggregation. For a given target node, we collect its partners whose labels are known. The frequency distribution of the node label types that are connected to the target node is then determined. We normalize by dividing by the total in or out strength of each frequency of node label, giving the discrete probability distribution of the in/out-connections to each of the labels. This removes biases of scale in the network, and addresses the in and out connections separately. The resulting vector has entries corresponding to proportion of contributions *in*-from or *out*-to each node label type.

Mathematically, let *a*_*iz*_ be the elements of an adjacency matrix, denoting the weight of a directed edge from neuron *i* to neuron *z*, with value zero if the edge does not exist. Let *b*_*zj*_ be another matrix element where *b*_*zj*_ = 1 if node *z* is labeled with annotation type *x*_*j*_ (e.g., a specific hemilineage) and zero otherwise. Then the vector of annotation type contributions for the in and out connectivity becomes:

$$\nu_{ij}^{\text{out}}=\sum_{z}a_{iz}b_{zj};\;\rho_{ij}^{\text{out}}=\frac{\nu_{ij}^{\text{out}}}{\sum_{j^{\prime }}\nu_{ij^{\prime }}^{\text{out}}},$$ (1)

and the in-vector is the same, with a transposed adjacency matrix element (*a*_*zi*_):

$$\nu_{ij}^{\text{in}}=\sum_{z}a_{zi}b_{zj};\;\rho_{ij}^{\text{in}}=\frac{\nu_{ij}^{\text{in}}}{\sum_{j^{\prime }}\nu_{ij^{\prime }}^{\text{in}}},$$ (2)

where the probability vectors (*ρ*) are normalized to sum to unity, and can write as $p_{i}^{\alpha }$, where *α* ∈ [in, out] can denote an in-vector or out-vector in reference to node *i*. For clarity, let’s drop *α* as the derivations are identical and discuss in and out directions later.

Notice that **p**_*i*_ is a probability distribution equivalent to *p*^(*i*)^(*X*), where *X* is a random variable that can take the values of the annotation types, *X* ∈ {*x*_1_, *x*_2_, …, *x*_*M*_}. We use the shorthand for probability for annotation type *x*_*j*_ as $p^{\left(i\right)}\left(X=x_{j}\right)=p_{j}^{\left(i\right)}$. Then, by using information entropy, we can measure specialization in this particular probability distribution:

$$S^{\left(i\right)}=-\sum_{j=1}^{N}p_{j}^{\left(i\right)}\ln\left(p_{j}^{\left(i\right)}\right).$$ (3)

Entropy is maximized when all contributions *p*_*j*_ are equal, meaning the node is connected to all types of labels equally so it is not specialized. Entropy is minimal when the connectivity is concentrated rather than distributed, with the minimum entropy value being 0 where all connections are with one label type. This means that specialized nodes have low entropy.

Now, if we have many probability vectors, we can calculate how specialized they are individually and so compare how diversely specialized the probability vectors are. Let *P* = {*p*^(1)^(*X*), *p*^(2)^(*X*), …, *p*^(*N*)^(*X*)} be a set of normalized connectivity probability distributions, where *N* is the number of distributions. Associated with the probability vectors, are each of their individual entropies: *S* = {*s*^(1)^, *s*^(2)^, …, *s*^(*N*)^}.

The average entropy is given by:

$$\bar{S}=\frac{1}{N}\sum_{i=1}^{N}s^{\left(i\right)}.$$ (4)

The average connectivity vector component $\bar{p}\left(X=x_{j}\right)$, written with a shorthand ${\bar{p}}_{j}$ is given by:

$${\bar{p}}_{j}=\frac{1}{N}\sum_{i}^{N}p^{\left(i\right)}\left(X=x_{j}\right)$$ (5)

which can be demonstrated that it is also a probability distribution with the property $\sum_{j}{\bar{p}}_{j}=1$ (see Supporting Information). Thus, the entropy of the average vector can be calculated and meaningfully compared:

$$S\left(\bar{p}\right)=-\sum_{j=1}^{M}{\bar{p}}_{j}\ln\left({\bar{p}}_{j}\right).$$ (6)

We are particularly interested in neurons are connected to other neurons with their own specific specialisms, which are individually diverse. If a given neuron’s connectivity is concentrated or specialized, then the entropy of this neuron will be low. If a group of neurons whose connectivity is specialized in the same node label types, then the average entropy, $\bar{S}$, will be low as well as the entropy of the average vector, $S\left(\bar{p}\right)$. If a group of neurons whose connectivity is specialized in different node label types, the average entropy, $\bar{S}$, will be low; however, the average vector will not be specialized so the entropy of the average vector, $S\left(\bar{p}\right)$, will be high. This is illustrated in Figure 1.

If the average entropy is high, the group of nodes are unspecialized so the average vector must also be unspecialized and the entropy of the average vector is also high. With this, we observe that large differences between the average entropy and the entropy of the average vector mean that the group of neurons have specialisms in a variety of different node label types. If the entropy averages’ differences are small, then it could mean that the group of neurons were not specialized to begin with, or that the group of neurons were all specialized in similar node label types (Figure 1A, top two rows).

Using these measures, we demonstrate that a high difference in entropy of the average vector and the average entropy of each vector is only observed when the set of vectors are diversely specialized (Figure 1A, bottom row). We arrive at the Δ*_S_*, the *average entropies’ differences*—the difference in the average entropy and the entropy of the average vector, which measures the *specialization-diversity*:

$$\Delta_{S}=S\left(\bar{p}\right)-\bar{S}.$$ (7)

Entropy is a concave function, so negative entropy is a convex function. This means that negative entropy must obey Jensen’s inequality for convex functions: $E\left[\phi \left(X\right)\right]-\phi \left(E\left(X\right)\right)\geq 0$, where E denotes the expected value and *ϕ* is a convex function on a probability distribution *X*. Therefore, substituting for −*S*(*p*) = *ϕ*(*X*), and $\bar{\cdot }$ for the expected value, $-\bar{S}+S\left(\bar{p}\right)\geq 0$, and so Δ*_S_* ≥ 0 always. This is logically sound, as a set of probability vectors would not become more specialized than the individual constituent vectors through averaging. In other words, the entropy of the average vector would always be at least value of the average entropy of those vectors.

Notice that we have two choices of the set of probability distributions *P* we use to calculate the specialization-diversity: which set of nodes *i* = 0, …, *N* to consider, and which direction the connectivity is aggregated (*α* ∈ [in, out] we dropped earlier). We are free to calculate a specialization-diversity for any combinations of upstream or downstream partners; input or output vectors. We focus here only on the upstream inputs (integrative) and downstream outputs (distributive), and label them $\Delta_{S}^{U,\text{in}}$ and $\Delta_{S}^{D,\text{out}}$, respectively. In other words, for a given target node, we may collect its upstream partners’ input vectors and we can calculate the specialization-diversity associated to this (Figure 1B, right). Secondly, we may use the downstream partners’ output vectors of a given node to calculate the specialization-diversity.

### *Drosophila* VNC and *C. elegans* Datasets

The *Drosophila* VNC connectome and associated annotations used in this paper is the Male Adult Nerve Cord data available from https://neuprint.janelia.org/ publically available with a Google account (see Takemura et al., 2024). The *C. elegans* connectome is a hermaphrodite data set from www.nemanode.org (Witvliet et al., 2021) by Varshney et al. (2011) (based on White et al., 1986) and the associated metadata was compiled from Cook et al. (2019) available from https://www.wormatlas.org/neuronalwiring.html, and Ripoll-Sánchez et al. (2023).

Annotations that were used in this paper for the connectivity vectorisation for specialization-diversity calculations were available from the data sources listed above. For *Drosophila*, we have hemilineage determined from light level data and morphology and connectivity clustering detailed in Marin et al. (2024). The secondary neurons of hemilineages have been experimentally demonstrated to form behavioral phenotypes in independent of brain signaling (Harris et al., 2015). Here, lineages are specified by neuronal precursor cells (ganglion mother cell), which then diverge via Notch signaling into two distinct phenotypes “A” and “B,” which form the hemilineages. These hemilineages are the developmental units of organization in *Drosophila* (Truman, Moats, Altman, Marin, & Williams, 2010). By calculating the specialization-diversities with the hemilineage annotation scheme, we are interested in how diversely specialized are the connectivity channels that perform these functions. In the Supporting Information, we investigate whether specialization-diversity averaged for each hemilineage reveals neurons that are highly important as they are proximal for collecting information from upstream and dispersing information downstream of many different functions.

In *C. elegans*, the annotations we use are mid-scale cell categorization by Cook et al. (2019), based on anatomy and information flow. This further splits interneurons into layers, specifying the sensory modality and motor location. These labels are directly associated with functionality, as opposed to the high-level cell classes (motor, sensory, interneuron and pharynx). The other alternative was to use the 118 cell types determined from transcriptomic profiles (Taylor et al., 2021). We deemed the high-level cell classes to be too broad, and the transcriptomic classes to be too specific. If the annotations are too fine, then the connectivity profiles may be too dissimilar between similar neurons; conversely, coarse-grained annotations are too general so the neighborhood vectors are too similar. Therefore, we utilized the mid-scale cell categories for constructing our annotation vectors. We implemented all null distribution tests with the finest cell types for the strictest test of correlation to known neurobiological criteria. These cell types are presented in Supporting Information Table S2. We tested alternative annotations (fine and coarse) in the vector construction for the specialization-diversity calculation, but did not offer as interpretable results for the above reasons. Exact determination of how optimal an annotation scheme is correlated to what interpretable property of the system is an open question.

## ACKNOWLEDGMENTS

We thank Gregory S. X. E. Jefferis, Philipp Schlegel, Tomke Stürner, and Elizabeth Marin for discussions and providing access to data.

## SUPPORTING INFORMATION

Supporting information for this article is available at https://doi.org/10.1162/NETN.a.533.

## AUTHOR CONTRIBUTIONS

Sung Soo Moon: Conceptualization; Data curation; Formal analysis; Investigation; Methodology; Software; Validation; Visualization; Writing – original draft; Writing – review & editing. Lidia Ripoll-Sánchez: Data curation; Formal analysis; Writing – review & editing. Petra Vértes: Supervision; Writing – review & editing. William R. Schafer: Supervision; Writing – review & editing. Sebastian E. Ahnert: Conceptualization; Formal analysis; Funding acquisition; Investigation; Methodology; Project administration; Resources; Supervision; Writing – review & editing.

## FUNDING INFORMATION

Sung Soo Moon., Harding Distinguished Postgraduate Scholars Programme Leverage Scheme and the Engineering and Physical Sciences Research Council Doctoral Training Partnerships (EPSRC DTP), Award ID: N/A.

## CODE AVAILABILITY

All files for python analysis for generating figures is documented and made available from https://github.com/sungsushi/spec_div. We include all data and processed data from this work in https://doi.org/10.5281/zenodo.15052465.

We utilise the scipy.stats.ks_2samp implementation of the two-sample Kolmogorov–Smirnov test.

## Supplementary Material



## TECHNICAL TERMS

Metadata: Informative categorical or continuous values assigned to nodes or edges that supplements the graph structure.
Local: Used to describe a quantity derived from a subgraph constructed around a particular node of interest in a larger network.
Information entropy: A statistical quantity to measure the average uncertainty of an outcome in a probability distribution.
Hemilineage: A set of neurons originating from one neuronal stem cell, representing one of the two diverging branches with shared ancestry and fate.
Pharynx: The enteric nervous system of *C. elegans*.
Neuropil: A region dense with axons, dendrites, and synapses that form functional neural circuits.
Klinotaxis: Movement in response to a chemical stimulus by sampling the intensity gradient of the stimulus.
