# Supplementary material for: An entropic measure of diverse specialization highlights multifunctional neurons in annotated connectomes
Source: Netw Neurosci. 2026 Mar 20;10(1):204–20. doi: 10.1162/NETN.a.533 (PMC13008378; doi:10.1162/NETN.a.533)
Supplement: Supplementary file 1 [file netn-10-1-204-s001.pdf]

# Supplementary Information for "An entropic measure of diverse specialisation highlights multifunctional neurons in annotated connectomes"

Sung Soo Moon, Lidia Ripoll-Sánchez, Petra Vértes,
William R. Schafer and Sebastian E. Ahnert

## Contents

**S1 Supplementary Information** **1**
S1.1 Proof that the category-wise mean of a set of discrete probability distributions is also a probability distribution . . . 1
S1.2 Alternate form for $\Delta_S$ . . . 2
S1.3 Bounds on $\Delta_S$ . . . 3
S1.4 Hemilineage functionality is reflected in specialisation-diversity distributions . . . 3

**S2 Table of cell classes in *C. elegans*** **5**

# S1 Supplementary Information

## S1.1 Proof that the category-wise mean of a set of discrete probability distributions is also a probability distribution

Let $P = \{p^{(1)}(X), p^{(2)}(X), ..., p^{(N)}(X)\}$ be a set of probability distributions, where $N$ is the number of distributions and $x$ is the discrete random variable $X \in \{x_1, x_2, ..., x_M\}$.

By definition, the sum of the $i$th probability distribution adds to one:

$$\sum_j^M p^{(i)}(X = x_j) = 1, \quad 0 \leq i \leq N, \quad 0 \leq j \leq M.$$

We take the category-wise mean of this set of probability distributions:

$$\bar{p}(X = x_j) = \frac{1}{N} \sum_i^N p^{(i)}(X = x_j)$$

which is the probability of $x$ averaged over all the distributions. $\bar{p}$ must be positive and bounded by the interval $[0, 1]$ since the constituent probability distributions are also positive and bounded for all $i, j$.

To check that this distribution indeed does sum to unity:

$$\sum_j^M \bar{p}(X = x_j) = \sum_j^M \left( \frac{1}{N} \sum_i^N p^{(i)}(X = x_j) \right) = \frac{1}{N} \sum_i^N \left( \sum_j^M p^{(i)}(X = x_j) \right) = \frac{1}{N} \sum_i^N 1 = 1$$

by using the previous definition of the probability vectors. Therefore, the category-wise mean of a set of discrete probability distributions is also a probability distribution.

## S1.2 Alternate form for $\Delta_S$

Here, we derive an alternate form for the specialisation-diversity which is helpful for implementation purposes and finding bounds on the metric.

Recall the definitions of $\bar{S}$, $S(\bar{p})$ and $\Delta_S$ in equations 4, 6 and 7 from the **Methods** section:

$$\bar{S} = \frac{1}{N}\sum_{i=1}^{N} s^{(i)}, \tag{4}$$

$$S(\bar{p}) = -\sum_{j=1}^{M} \bar{p}_j \ln(\bar{p}_j), \tag{6}$$

$$\Delta_S = S(\bar{p}) - \bar{S}. \tag{7}$$

Now, by substituting each term, we get:

$$\Delta_S = -\sum_{j=1}^{M} \bar{p}_j \ln(\bar{p}_j) - \frac{1}{N}\sum_{i=1}^{N} s^{(i)} = -\sum_{j=1}^{M}\left(\frac{1}{N}\sum_{l=1}^{N} p_j^{(l)}\right)\ln\left(\frac{1}{N}\sum_{q=1}^{N} p_j^{(q)}\right) + \frac{1}{N}\sum_{i=1}^{N}\sum_{k=1}^{M} p_k^{(i)}\ln(p_k^{(i)}). \tag{S1}$$

The first term can be simplified as follows:

$$-\sum_{j=1}^{M}\left(\frac{1}{N}\sum_{l=1}^{N} p_j^{(l)}\right)\ln\left(\frac{1}{N}\sum_{q=1}^{N} p_j^{(q)}\right) = \frac{-1}{N}\sum_{l=1}^{N}\sum_{j=1}^{M} p_j^{(l)}\left[-\ln(N) + \ln\left(\sum_{q=1}^{N} p_j^{(q)}\right)\right] \tag{S2}$$

$$= \frac{\ln(N)}{N}\sum_{l=1}^{N}\sum_{j=1}^{M} p_j^{(l)} - \frac{1}{N}\sum_{l=1}^{N}\sum_{j=1}^{M} p_j^{(l)}\ln\left(\sum_{q=1}^{N} p_j^{(q)}\right) = \ln(N) - \frac{1}{N}\sum_{l=1}^{N}\sum_{j=1}^{M} p_j^{(l)}\ln\left(\sum_{q=1}^{N} p_j^{(q)}\right) \tag{S3}$$

where we invoke that the category-wise average of a probability distribution is itself a probability distribution and must sum to one.

Now, substituting this back to equation S1 :

$$\Delta_S = \ln(N) - \frac{1}{N}\sum_{l=1}^{N}\sum_{j=1}^{M} p_j^{(l)}\ln\left(\sum_{q=1}^{N} p_j^{(q)}\right) + \frac{1}{N}\sum_{i=1}^{N}\sum_{k=1}^{M} p_k^{(i)}\ln(p_k^{(i)}), \tag{S4}$$

we may rewrite the sums over the labels and the sums over the vectors (i.e. setting $j = k$ and $i = l$)

$$\Delta_S = \ln(N) + \frac{1}{N}\sum_{i=1}^{N}\sum_{j=1}^{M}\left[-p_j^{(i)}\ln\left(\sum_{q=1}^{N} p_j^{(q)}\right) + p_j^{(i)}\ln(p_j^{(i)})\right]. \tag{S5}$$

Then, writing it as one logarithm:

$$\Delta_S = \ln(N) + \frac{1}{N}\sum_{i=1}^{N}\sum_{j=1}^{M} p_j^{(i)}\ln\left[\frac{p_j^{(i)}}{\sum_{q=1}^{N} p_j^{(q)}}\right]. \tag{S6}$$

We consider $p_j^{(i)}$ to be the probability of label $j$ given that the distribution is the $i$th vector, and can write as a conditional probability: $p(j|i)$. And so in this interpretation, the term

$$\frac{p_j^{(i)}}{\sum_{q=1}^{N} p_j^{(q)}} = p(i|j), \tag{S7}$$

**Table S1:** Hemilineages and their link to motor functions in the VNC in the absence of brain signalling from Harris et al., 2015

| **Function** | **Hemilineages** |
|---|---|
| Posture | 03B, 23B, 12B, 13B, 05B, 20A.22A, 08A, 09A |
| Uncoordinated leg movement (`uc_leg_movement`) | 06A, 06B, 19A, 21A, 23B |
| Walking | 18B, 12A, 01A, 10B |
| Wing wave | 12A, 03B |
| Wing buzz | 07B, 11A, 11B, 02A, 18B, 12A |
| Take-off | 07B, 11A, 11B, 18B |

as it is the probability of choosing vector $i$ given that we consider label $j$. The notation is consistent with conditional probability obeying Bayes' rule.

Finally, we arrive at:

$$\Delta_S = \ln(N) + \frac{1}{N}\sum_{i=1}^{N}\sum_{j=1}^{M} p(j|i)\ln(p(i|j)). \tag{S8}$$

## S1.3 Bounds on $\Delta_S$

The lower bound for specialisation-diversity is zero due to Jensen's inequality. For an upper bound, consider equation S8. The second term is always negative since $p(j|i)\ln(p(i|j)) \leq 0$ as $0 \leq p(i|j) \leq 1$. (We make use of the limit $\lim_{x\to 0} x\ln(x) \to 0$, and by definition in equation S7, $p(j|i) = 0$ if $p(i|j) = 0$.) Consider the case where we have fewer vectors than categories, $N < M$. $\Delta_S$ is maximised when $p(j|i)\ln(p(i|j)) = 0$ for all pairs of $i, j$. Physically, this would mean maximally specialised ($p(i|j) = p(j|i) = 1$) in different categories, and the remaining entries being zero ($p(i|j) = p(j|i) = 0$). And so the maximum value for $\Delta_S$ is $\ln(N)$. When there are more vectors than categories ($N > M$), we must have a degeneracy in specialism even in the most diversely specialised scenario, thus imposes a stricter bound $\Delta_S < \ln(N)$. This would contribute to degree hubs (large $N$ compared to $M$, the number of annotation types) being penalised in their specialisation-diversity value if their partners are degenerately specialised.

## S1.4 Hemilineage functionality is reflected in specialisation-diversity distributions

From Harris et al., 2015, we know which *Drosophila* hemilineages (secondary neurons) activate when certain functions are observed. These are: changes in posture, uncoordinated leg movement, walking, wing wave, wing buzz and take-off, and many hemilineages were activiated in more than one functions. We binarised these relationships, and sought to ask are the complexities in functionality, which is summarised in table S1.

When we averaged the integrative and distributive specialisation-diversities we found that even with the 'same' functional were very different in specialisation-diversity values (figure S1), suggesting that the functional categorisation may offer itself to further sub-roles that require different levels of diversely specialised connectivity channels.

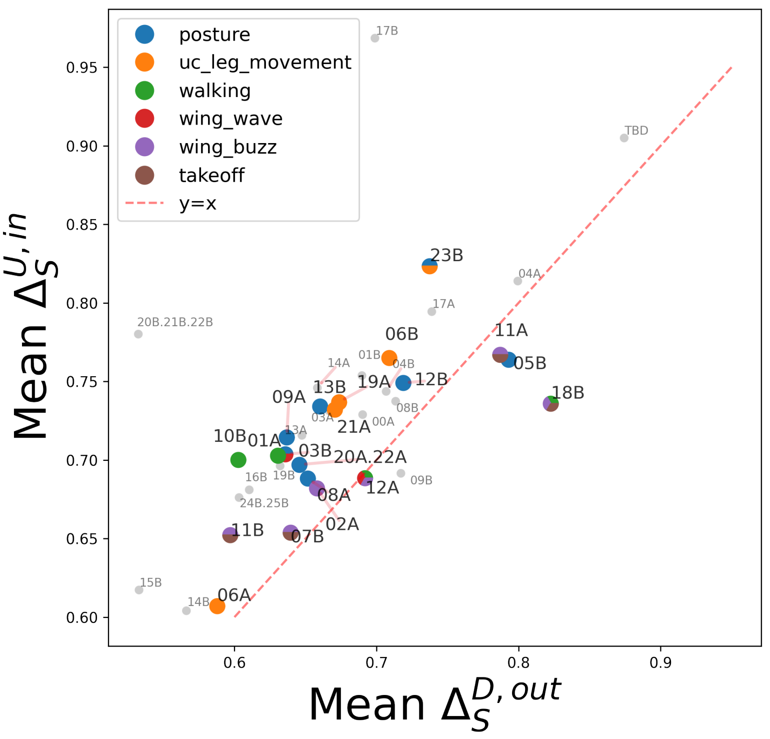


**Figure S1: Hemilineages with same function may have more specialised roles**
Here, we plot the mean integrative vs distributive specialisation-diversities averaged across all secondary birthtime (post-embryonic) neurons of each hemilineage in the *Drosophila* VNC. We note each function by six colours in the legend, and split the marker colours when a hemilineage is associated to multiple functions. In grey are hemilineages not described in Harris et al., 2015. The red dotted $y = x$ line is where points are equally distributive and integrative.

## S2 Table of cell classes in *C. elegans*

We show the coarse, mid-scale and fine annotations associated with the *C. elegans* neurons in table S2, see **Methods** for details.

**Table S2:** Three neuronal classifications of various coarseness are given. The coarsest cell class is from Ripoll-Sánchez et al., 2023, "Final Classification", the intermediary mid-scale cell category used to construct the annotation vectors is from Cook et al., 2019 under the name "cell category" and the finest types are the canonical 118 numerical "cell class" labels from Ripoll-Sánchez et al., 2023, compiled from morphological, transcriptomic and connectivity characterisation Hobert et al., 2016; Taylor et al., 2021.

| Coarse | Mid-scale | Fine | Neuron |
|---|---|---|---|
| pharynx | ph_interneuron | 1 | I1L, I1R |
| | | 14 | NSML, NSMR |
| | | 2 | I2L, I2R |
| | | 3 | I3 |
| | | 4 | I4 |
| | | 5 | I5 |
| | | 6 | I6 |
| | ph_motorneuron | 10 | M4 |
| | | 11 | M5 |
| | | 12 | MCL, MCR |
| | | 13 | MI |
| | | 7 | M1 |
| | | 8 | M2L, M2R |
| | | 9 | M3L, M3R |
| interneuron | category 4 interneuron | 58 | AIML, AIMR |
| | | 59 | AINL, AINR |
| | | 69 | RIH |
| | | 70 | RIR |
| | layer 1 interneuron | 64 | RIAL, RIAR |
| | | 87 | RID |
| | | 88 | PVCL, PVCR |
| | | 89 | AVAL, AVAR |
| | | 90 | AVBL, AVBR |
| | | 92 | AVEL, AVER |
| | | 96 | RIML, RIMR |
| | layer 2 interneuron | 54 | AIBL, AIBR |
| | | 65 | RIBL, RIBR |
| | | 66 | RICL, RICR |
| | | 68 | RIGL, RIGR |
| | | 72 | RMGL, RMGR |
| | | 76 | AVJL, AVJR |
| | | 77 | AVKL, AVKR |
| | | 78 | AVL |
| | | 80 | DVC |
| | | 85 | PVT |
| | | 86 | PVWL, PVWR |
| | | 91 | AVDL, AVDR |
| | | 94 | SAADL, SAADR, SAAVL, SAAVR |
| | layer 3 interneuron | 52 | AUAL, AUAR |
| | | 53 | AIAL, AIAR |
| | | 55 | AIYL, AIYR |
| | | 56 | AIZL, AIZR |
| | | 57 | ADAL, ADAR |
| | | 60 | ALA |
| | | 61 | BDUL, BDUR |
| | | 67 | RIFL, RIFR |
| | | 71 | RIS |
| | | 73 | AVFL, AVFR |
| | | 74 | AVG |
| | | 75 | AVHL, AVHR |
| | | 79 | DVB |
| | | 81 | PVNL, PVNR |
| | | 82 | PVPL, PVPR |
| | | 83 | PVQL, PVQR |
| | | 84 | PVR |
| | | 93 | LUAL, LUAR |
| | linker to pharynx | 63 | RIPL, RIPR |
| | sublateral motor neuron | 95 | SABD, SABVL, SABVR |
| motor neuron | SN1 | 97 | IL1DL, IL1DR, IL1L, IL1R, IL1VL, IL1VR |
| | head motor neuron | 100 | RMDDL, RMDDR, RMDL, RMDR, RMDVL, RMDVR |
| | | 101 | RMED, RMEL, RMER, RMEV |
| | | 103 | RMHL, RMHR |
| | | 104 | RIVL, RIVR |
| | | 107 | URADL, URADR, URAVL, URAVR |
| | layer 2 interneuron | 102 | RMFL, RMFR |
| | sex-specific neuron | 117 | HSNL, HSNR |

Continued on next page

**Table S2 – continued from previous page**

| Coarse cell type | Mid-scale cell category | Fine cell class | Neuron |
|---|---|---|---|
| | | 118 | VC01, VC02, VC03, VC04, VC05, VC06 |
| | sublateral motor neuron | 105 | SMBDL, SMBDR, SMBVL, SMBVR |
| | | 106 | SMDDL, SMDDR, SMDVL, SMDVR |
| | | 98 | SIADL, SIADR, SIAVL, SIAVR |
| | | 99 | SIBDL, SIBDR, SIBVL, SIBVR |
| | ventral cord motor neuron | 108 | DA01, DA02, DA03, DA04, DA05, DA06, DA07, DA08, DA09 |
| | | 109 | PDA |
| | | 110 | DB01, DB02, DB03, DB04, DB05, DB06, DB07 |
| | | 111 | AS01, AS02, AS03, AS04, AS05, AS06, AS07, AS08, AS09, AS10, AS11 |
| | | 112 | PDB |
| | | 113 | DD01, DD02, DD03, DD04, DD05, DD06 |
| | | 114 | VA01, VA02, VA03, VA04, VA05, VA06, VA07, VA08, VA09, VA10, VA11, VA12 |
| | | 115 | VB01, VB02, VB03, VB04, VB05, VB06, VB07, VB08, VB09, VB10, VB11 |
| | | 116 | VD01, VD02, VD03, VD04, VD05, VD06, VD07, VD08, VD09, VD10, VD11, VD12, VD13 |
| sensory neuron | SN1 | 27 | IL2DL, IL2DR, IL2L, IL2R, IL2VL, IL2VR |
| | | 28 | OLQDL, OLQDR, OLQVL, OLQVR |
| | | 29 | OLLL, OLLR |
| | | 30 | CEPDL, CEPDR, CEPVL, CEPVR |
| | | 41 | URYDL, URYDR, URYVL, URYVR |
| | SN2 | 46 | PHAL, PHAR |
| | | 47 | PHBL, PHBR |
| | | 48 | PHCL, PHCR |
| | SN3 | 31 | ALML, ALMR |
| | | 32 | AVM |
| | | 33 | PVM |
| | | 34 | PLML, PLMR |
| | | 37 | FLPL, FLPR |
| | | 38 | PVDL, PVDR |
| | | 44 | ADEL, ADER |
| | | 45 | PDEL, PDER |
| | | 51 | DVA |
| | SN4 | 35 | ALNL, ALNR |
| | | 36 | PLNL, PLNR |
| | | 39 | BAGL, BAGR |
| | | 40 | URXL, URXR |
| | | 42 | AQR |
| | | 43 | PQR |
| | | 49 | SDQL, SDQR |
| | SN5 | 16 | ADLL, ADLR |
| | | 19 | ASHL, ASHR |
| | SN6 | 15 | ADFL, ADFR |
| | | 17 | ASEL, ASER |
| | | 18 | ASGL, ASGR |
| | | 20 | ASIL, ASIR |
| | | 21 | ASJL, ASJR |
| | | 22 | ASKL, ASKR |
| | | 23 | AWAL, AWAR |
| | | 24 | AWBL, AWBR |
| | | 25 | AWCL, AWCR |
| | | 26 | AFDL, AFDR |
| | category 4 interneuron | 50 | URBL, URBR |
| unknown | endorgan | 62 | CANL, CANR |

# References


Cook, S. J., Jarrell, T. A., Brittin, C. A., Wang, Y., Bloniarz, A. E., Yakovlev, M. A., Nguyen, K. C. Q., Tang, L. T.-H., Bayer, E. A., Duerr, J. S., Bülow, H. E., Hobert, O., Hall, D. H., & Emmons, S. W. (2019). Whole-animal connectomes of both Caenorhabditis elegans sexes [Publisher: Nature Publishing Group]. *Nature*, *571*(7763), 63–71. https://doi.org/10.1038/s41586-019-1352-7

Harris, R. M., Pfeiffer, B. D., Rubin, G. M., & Truman, J. W. (2015). Neuron hemilineages provide the functional ground plan for the Drosophila ventral nervous system (L. C. Griffith, Ed.) [Publisher: eLife Sciences Publications, Ltd]. *eLife*, *4*, e04493. https://doi.org/10.7554/eLife.04493

Hobert, O., Glenwinkel, L., & White, J. (2016). Revisiting Neuronal Cell Type Classification in *Caenorhabditis elegans*. *Current Biology*, *26*(22), R1197–R1203. https://doi.org/10.1016/j.cub.2016.10.027

Ripoll-Sánchez, L., Watteyne, J., Sun, H., Fernandez, R., Taylor, S. R., Weinreb, A., Bentley, B. L., Hammarlund, M., Miller, D. M., Hobert, O., Beets, I., Vértes, P. E., & Schafer, W. R. (2023). The neuropeptidergic connectome of *C. elegans*. *Neuron*, *111*(22), 3570–3589.e5. https://doi.org/10.1016/j.neuron.2023.09.043

Taylor, S. R., Santpere, G., Weinreb, A., Barrett, A., Reilly, M. B., Xu, C., Varol, E., Oikonomou, P., Glenwinkel, L., McWhirter, R., et al. (2021). Molecular topography of an entire nervous system. *Cell*, *184*(16), 4329–4347.
